# Elucidating causative gene variants in hereditary Parkinson’s disease in the Global Parkinson’s Genetics Program (GP2)

**DOI:** 10.1038/s41531-023-00526-9

**Published:** 2023-06-27

**Authors:** Lara M. Lange, Micol Avenali, Melina Ellis, Anastasia Illarionova, Ignacio J. Keller Sarmiento, Ai-Huey Tan, Harutyun Madoev, Caterina Galandra, Johanna Junker, Karisha Roopnarain, Justin Solle, Claire Wegel, Zih-Hua Fang, Peter Heutink, Kishore R. Kumar, Shen-Yang Lim, Enza Maria Valente, Mike Nalls, Cornelis Blauwendraat, Andrew Singleton, Niccolo Mencacci, Katja Lohmann, Christine Klein, the Global Parkinson’s Genetic Program (GP2)

**Affiliations:** 1 Institute of Neurogenetics, University of Lübeck, Lübeck, Germany; 2 IRCCS Mondino Foundation, Pavia, Italy; 3 Department of Brain and Behavioral Sciences, University of Pavia, Pavia, Italy; 4 Northcott Neuroscience Laboratory, ANZAC Research Institute, Concord, NSW Australia; 5 Faculty of Medicine and Health, University of Sydney, Sydney, NSW Australia; 6 German Center for Neurodegenerative Diseases (DZNE), Tübingen, Germany; 7 Department of Neurology, Northwestern University Feinberg School of Medicine, Chicago, IL USA; 8 Division of Neurology, Department of Medicine, and the Mah Pooi Soo and Tan Chin Nam Centre for Parkinson’s and Related Disorders, Faculty of Medicine, University of Malaya, Kuala Lumpur, Malaysia; 9 Department of Molecular Medicine, University of Pavia, Pavia, Italy; 10 Department of Clinical Research, Michael J. Fox Foundation for Parkinson’s Research, New York City, NY USA; 11 Department of Medical and Molecular Genetics, Indiana University School of Medicine, Indianapolis, IN USA; 12 Garvan Institute of Medical Research, Darlinghurst, NSW Australia; 13 Molecular Medicine Laboratory and Neurology Department, Concord Repatriation General Hospital, The University of Sydney, Concord, NSW Australia; 14 Data Tecnica International, Washington, DC USA; 15 Center for Alzheimer’s and Related Dementias (CARD), National Institute on Aging and National Institute of Neurological Disorders and Stroke, National Institutes of Health, Bethesda, MD USA; 16 Molecular Genetics Section, Laboratory of Neurogenetics, National Institute on Aging, National Institutes of Health, Bethesda, MD USA; 17 Integrative Genomics Unit, Laboratory of Neurogenetics, National Institute on Aging, National Institutes of Health, Bethesda, MD USA

**Keywords:** Sequencing, Parkinson's disease

## Abstract

The Monogenic Network of the Global Parkinson’s Genetics Program (GP2) aims to create an efficient infrastructure to accelerate the identification of novel genetic causes of Parkinson’s disease (PD) and to improve our understanding of already identified genetic causes, such as reduced penetrance and variable clinical expressivity of known disease-causing variants. We aim to perform short- and long-read whole-genome sequencing for up to 10,000 patients with parkinsonism. Important features of this project are global involvement and focusing on historically underrepresented populations.

The Global Parkinson’s Genetics Program (GP2, http://gp2.org/) is an international collaborative effort that aims to substantially improve our understanding of the role that genetics plays in Parkinson’s disease (PD) and to make this knowledge globally available and actionable^1^. The Monogenic Network of GP2 creates an efficient infrastructure to accelerate the identification of novel monogenic causes of parkinsonism but also to improve our current understanding of known genetic causes, such as reduced penetrance and variable clinical expressivity of known genetic variants. There will also be a corresponding manuscript from the Complex Disease Network of GP2 focusing on sporadic PD patients and controls and investigating complex genetic causes of the disease. To address a large gap in our knowledge of PD due to the lack of genetic studies in diverse populations, we bring together clinicians, researchers and existing PD consortia from around the globe with a particular emphasis on historically underrepresented populations. We collect and generate clinical and genetic data of PD patients and families, harmonize and democratize data as well as the access to it, and develop analytical resources. All clinicians and researchers interested in collaborative PD genetics research are welcome to be part of the GP2 Monogenic Network and share data and biomaterials of PD patients and family members with (i) a known monogenic cause of disease or (ii) an unknown but suspected monogenic cause, e.g., based on a young age at onset (AAO) or a positive family history.

The workflow of the Monogenic Network of GP2 is shown in Fig. 1. The first step is to register to the Monogenic Portal (https://monogenic.gp2.org/monogenicportal.html). This online platform serves as a secure bidirectional site to upload and download clinical and genetic data regarding patients/families with potentially monogenic PD. More specifically, the Portal allows participants to readily access information on the project, facilitates the collection of detailed data, and enables a deeper analysis of genetic, clinical-demographic, and environmental factors influencing PD development and expression^2,3^. Key items include a registration site to submit information regarding institutional ethical clearance for international data and sample sharing and an electronic case report form (eCRF) to submit pseudonymized data of patients/families securely. As a prerequisite, every research institution has to obtain institutional ethics approval. Researchers are required to submit their ethics documents through the Portal for GP2 compliance review. Following compliance approval, research agreements will be initiated before data and samples can be shared.

**Fig. 1:**
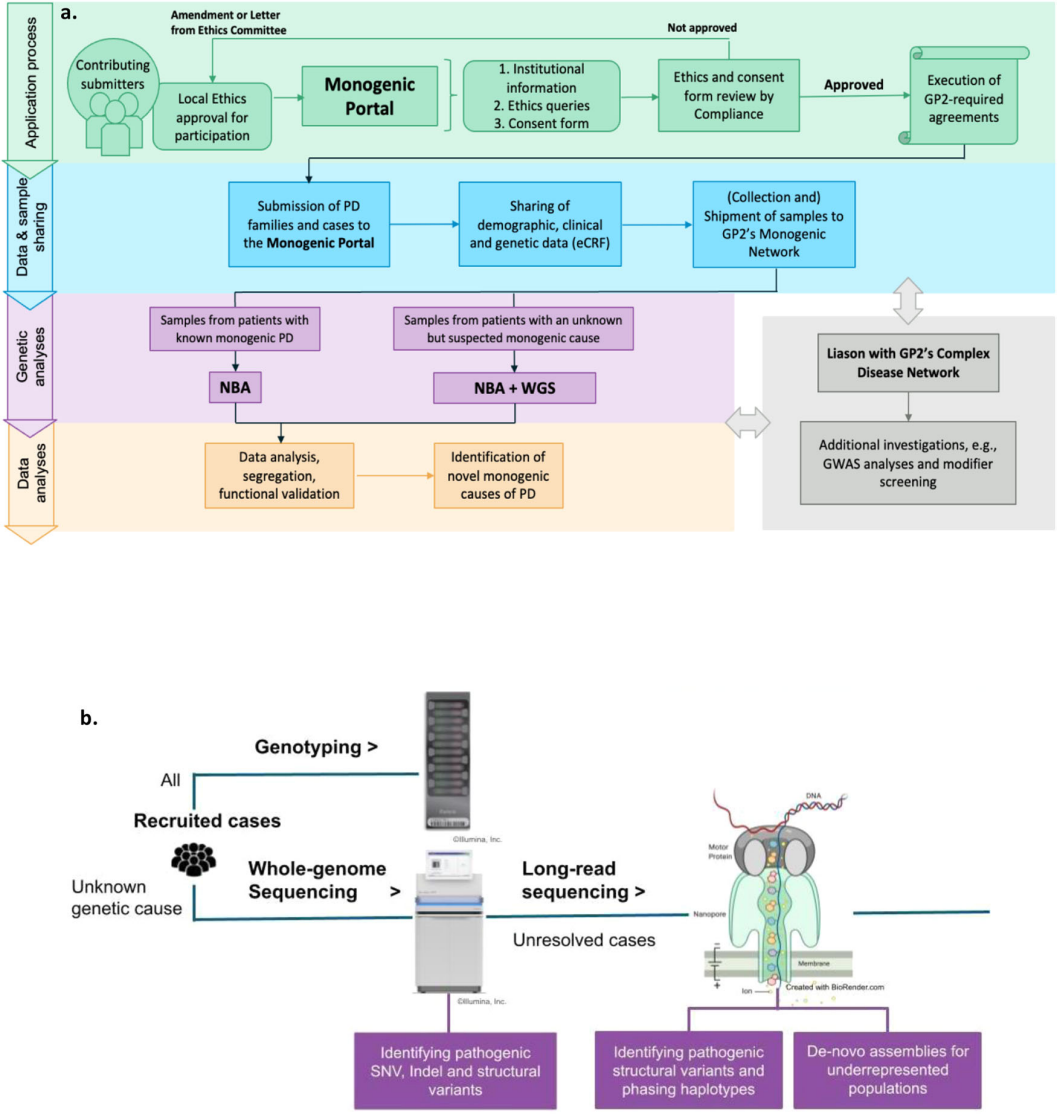
Workflow of the GP2 Monogenic Network. Figure 1 displays the general workflow of GP2’s Monogenic Network (**a**) and the specific data generation and processing workflow (**b**). **a** There are four steps: (I) The application process, where interested collaborators register to the Monogenic Portal, ethics documents are reviewed, and the required paperwork is executed. (II) The data and sample sharing, where collaborators ship their samples to GP2’s Monogenic Network and share the respective clinical data for these patients by filling out the eCRFs in the Portal. (III) The genetic analyses that are performed by GP2’s Monogenic Network, in particular, NBA genotyping for all patients and both NBA and WGS for a subset of unsolved, apparently monogenic cases (for details, see **b**). (IV) The data analyses where generated data are interpreted, and results validated. **b** All samples from recruited cases will be genotyped with the Illumina NeuroBooster Array. Prioritized samples with an unsolved genetic cause will be whole-genome sequenced (WGS). Additionally, a subset of samples still unsolved after short-read WGS will undergo long-read sequencing. eCRF electronic case report form, GP2 Global Parkinson’s Genetics Program, GWAS genome-wide association studies, NBA NeuroBooster Array, PD Parkinson’s disease, WGS whole-genome sequencing.

The eCRF (https://monogenic.gp2.org/testing/ecrf1) consists of multiple questionnaires focusing on (i) demographics and basic clinical details, (ii) family history, (iii) PD clinical features, investigations, and treatments, and (iv) relevant environmental or acquired factors prior to PD motor symptom onset. Within the Portal, collaborators also gain access to research-based genetic results regarding their own cases and monitor the genetic analysis process.

All samples submitted to the Monogenic Network undergo quality control and are adequately prepared for genotyping and sequencing. The preferred sample type is high-quality genomic DNA (gDNA) obtained from blood or alternatively saliva, but fresh blood in an EDTA tube (10 ml), and frozen EDTA blood or saliva can also be accepted.

The Monogenic Network of GP2 focuses on monogenic causes of the disease and aims to identify and collect cases with a higher probability of finding novel PD-causing genes (criteria are listed in Supplementary Table 1). Priority is given to families with a greater number of available samples, given that this is likely to improve the filtering procedure of thousands of genetic variants from WGS data. We also prioritize consanguineous families which have a higher chance of carrying homozygous or compound-heterozygous recessive genetic variants. Also, a younger AAO of PD is prioritized given the increased genetic load in early-onset PD^4^. Furthermore, we preferentially include samples from underrepresented populations to support our goal of greater representation of these groups^5^. Additionally, the Monogenic Network is also interested in patients and families with genetic variants in already known PD genes.

Regardless of whether samples have been genetically tested before and whether they are already known to carry PD-related mutations, every sample will undergo genotyping with the NeuroBooster Array (NBA). This array includes 1.9 million markers from the Illumina Global Diversity Array and more than 95,000 neurological disease-oriented and population-specific variants, including several hundred known mutations in PD-related genes (https://github.com/GP2code/Neuro_Booster_Array). We will also test for large deletions and duplications encompassing known PD genes, which represent another frequent cause of monogenic PD^6,7^. Second, about 10,000 prioritized cases negative for mutations in known genes will undergo Illumina short-read WGS; the genetic workflow is displayed in Fig. 1b. We use the functional equivalence pipeline^8^ implemented at the Broad Institute to produce alignments and small variant calls against the GRCh38DH reference genome, as well as the Broad Institute’s joint discovery pipeline to produce a set of joint-genotyped variants for all the samples that pass WGS quality controls following the quality metrics defined by the Accelerating Medicines Partnership Parkinson’s Disease program (AMP-PD; https://amp-pd.org)^9^. After joint-genotyping, we retain high-quality variants that are flagged as PASS after variant quality score recalibration, with a call rate >0.95, genotype quality >20, read depth >5, and heterozygous allele balance between 0.25 and 0.75. Additionally, we discover large structural variants ( > 50 base pairs) for each sample using Parliament2 pipeline^10^ and perform joint-genotyping using graphtyper2^11^. Following variant quality control, we annotate variants with Ensembl Variant Effect Predictor^12^ to prioritize candidate single-nucleotide variants, indels, and structural variants based on population frequencies, segregation, and in silico predictor scores. All the pipelines and scripts used for monogenic data analyses are available via GitHub (https://github.com/GP2code/GP2-WorkingGroups/tree/main/MN-DAWG-Monogenic-Data-Analysis). In a third step, a subset of prioritized unsolved cases (*n* = 1000) will undergo long-read WGS with Oxford Nanopore technologies. Long-read sequencing has the capacity to sequence much longer reads compared to short-read (on average, over 10 kb in one single read). This method will be used to generate population-specific genome assembly, haplotype phasing, and the detection of repeat expansions and structural variants. In addition, we will integrate the resulting genetic data with the clinical information available from the Portal (e.g., AAO and clinical features).

To date, the Monogenic Network has contacted ~250 potential contributors from >60 different countries (Fig. 2). We are about to complete a 500-short-read WGS pilot project including 16 research teams from 10 countries covering five continents. Around three quarters of this pilot cohort were familial cases, whereas the remaining patients were singleton cases with an early age at onset of disease (≤40 years); twelve of these singletons were included as parent-offspring trios (index patient plus both clinically unaffected parents). Notably, ~20% of selected patients came from underrepresented populations, mainly South America and South-East Asia. Following the pilot, another ~2000 samples have already been submitted to the Monogenic Network and are currently undergoing genotyping and sequencing.

**Fig. 2:**
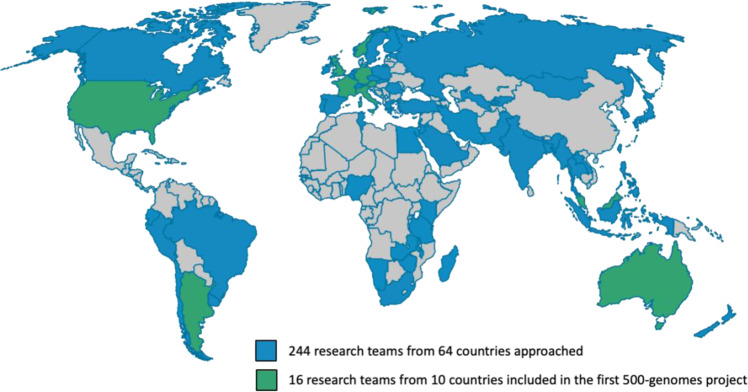
Outreach of the Monogenic Network (world map) and selected cohort for the 500-genomes pilot project (September 2022). Highlighted in blue are countries where potential collaborators have been contacted, and highlighted in green are the countries from which research teams have been involved in the 500-genomes pilot project. Over 60 of these teams from almost 40 countries are already participating in the GP2 project.

Hurdles encountered during the project so far include the administrative burden of ensuring compliance with ethics and local and international regulatory requirements. Furthermore, the SARS-CoV-2 pandemic has posed unique challenges by limiting personal visits and slowing recruitment. Moreover, there is a strong focus on recruiting and supporting participants from low-middle income countries that often have only limited experience, resources, and infrastructure for sample collection and processing.

Other important aspects to consider in such a global project are the extent of involvement and the respective credit for collaborating centers, particularly when it comes to analyzing the data and publishing the results. This should, of course, be addressed on a case-by-case basis, also depending on the expertize and goals of the collaborating center. There are different scenarios, all of which include the general GP2 pipeline as described above as the initial analysis step. Afterwards, sample submitters are free to work with the data generated from their samples, perform their own analyses, and publish their results. Genetic analyses can also be performed together with or solely by the GP2 data analysis teams depending on the experience and interest of the sample providers. Additional information on GP2’s privacy and open access policies can be found on the website (https://gp2.org). Work including GP2-generated data shall include a banner author list including all official members of GP2; more details in terms of authorship of potential abstracts or manuscripts have to be decided on an individual level. At its core, GP2 is a global endeavor that can only succeed through long term collaboration and cooperation, therefore it is fundamentally important that we ensure appropriate control, participation, and credit for all members.

The identification of genes causing monogenic PD has provided critical insights into the underlying disease pathophysiology. However, despite extensive investigations, only a minority (10–40%^13^) of likely monogenic cases receive a genetic diagnosis to date; it is, thus, imperative to investigate these unsolved families for novel PD genes. Moreover, even genetically proven cases entail significant challenges in terms of understanding the genetic modifiers and possible other additional factors underlying the variable penetrance and expressivity of many genetic mutations (e.g., *LRRK2* p.G2019S or *GBA1* variants). Understanding these modifying factors is likely to provide insights into disease pathogenesis and the potential routes to new treatment. Within the Monogenic Network of GP2, we have established a workflow that allows us to work together with clinicians and researchers from around the globe, overcome national and international privacy challenges, as well as obtain DNA samples and clinical information from likely and known monogenic PD cases, and perform high throughput genetic analysis. Given the extensive infrastructure and global outreach, this project is expected to contribute substantially to our understanding of the genetic basis of monogenic PD, with a particular focus on populations that have not yet been largely addressed by existing research.

## Methods

### Cohort recruitment

Researchers and clinicians have been contacted through personalized invitation emails or personal contacts. Interested PD clinicians and researchers will be initiated via introductory video conference calls covering the nature of GP2 and its goals, benefits of participation, sample and clinical data requirements, and ethical and compliance issues. If required, templates for research protocols, patient information, and consent forms are shared as needed, and assistance is provided for contributors who require help in their ethics application process.

### Ethics and consent

In order to participate, each collaborating site has to obtain approval from their local Ethics Committee, which will be reviewed by the Operations and Compliance Working Group (OCWG) of GP2. A list of all participating sites can be found on the GP2 website (https://gp2.org/cohort-dashboard-advanced/). Additionally, sample providers have to share their consent documents which are also reviewed by the OCWG of GP2 to ensure that international sample and data sharing is allowed and that local data sharing restrictions are respected. Written informed consent is obtained at each individual site according to the local ethics protocol approved by the OCWG. The OCWG also assists in case amendments or revisions are needed, and additional resources are provided online (https://gp2.org/resources/consent-guidelines/) or by working group members if needed.

Detailed information can also be found on the GP2 (https://gp2.org) and the Monogenic Network (https://monogenic.gp2.org/index.html) website.

### Sample requirements and preparation

The Monogenic Network accepts three different sample types:

1. The preferred sample type is high-quality genomic DNA from blood (alternatively from saliva)

- Required volume: >60 µl
- Required concentration: >50 ng/µl (in TE buffer)
- Quality: OD 260/280 nm: ~1.8

Shipping Instructions: submit sample in a clearly 9abelled 1.5–2.0 ml microcentrifuge tube sealed with parafilm tightly. Place sealed microcentrifuge tubes in a 50 ml disposable screw cap tube or a small solid box for additional insulation during shipment.

2. Alternatively, fresh ~10 ml EDTA blood.
  Collecting and shipping instructions: the tubes should be filled properly, inverted (not shaken) 10 times carefully. After collection, the tubes should be kept at 4 °C (do not freeze) and sent off as soon as possible. Place the EDTA tube in a 50 ml disposable screw cap tube or a small solid box for additional insulation during shipment. To prevent the EDTA tube from moving during shipment, fill any remaining space in the 50 ml tube or box with clean tissue paper or bubble wrap before sealing.
3. If you neither have isolated DNA nor can freshly collect a sample, we can also accept (although not preferred) frozen EDTA blood (~5 ml) or a frozen saliva sample. In this case, the blood or saliva has to be shipped on dry ice.

*Shipping Instructions:* Place the frozen EDTA/saliva tube in a 50 ml precooled, disposable screw cap tube or a small solid box for additional insulation during shipment.

Every DNA sample that is shared with the Monogenic Hub undergoes quality control. If samples do not meet the criteria, they are adjusted accordingly by either sample dilution with high-performance liquid chromatography (HPLC) water or by sample concentration by evaporation using a thermoshaker at 55 °C. Quality is checked with a spectrophotometer (Nanodrop^TM^1000 Spectrophotometer). When whole blood or saliva is submitted, DNA extraction is performed following standard techniques. For DNA extraction from whole blood (fresh or frozen), the Roche High Pure Viral Nucleic Acid Large Volume Kit is used, and for frozen saliva, the Norgen Saliva DNA Isolation Kit is used. In addition, an extraction machine, AGFSTAR (AutoGen), is used for large amounts of either fresh or frozen blood, together with the FlexiGene DNA extraction Kit (Qiagen). After sample preparation, DNA samples are pipetted into tubes and sent for NBA genotyping and/or WGS.

### Supplementary information

Supplementary Material

## Data Availability

GP2 has partnered with the Accelerating Medicines Partnership - Parkinson’s Disease (AMP-PD; https://amp-pd.org) to share data generated by GP2. The first GP2 data was released to the AMP-PD platform in December 2021, and there will be data releases at regular intervals as the project continues.
