# Author Correction: Elucidating causative gene variants in hereditary Parkinson’s disease in the Global Parkinson’s Genetics Program (GP2)

**DOI:** 10.1038/s41531-023-00560-7

**Published:** 2023-09-13

**Authors:** Lara M. Lange, Micol Avenali, Melina Ellis, Anastasia Illarionova, Ignacio J. Keller Sarmiento, Ai-Huey Tan, Harutyun Madoev, Caterina Galandra, Johanna Junker, Karisha Roopnarain, Justin Solle, Claire Wegel, Zih-Hua Fang, Peter Heutink, Kishore R. Kumar, Shen-Yang Lim, Enza Maria Valente, Mike Nalls, Cornelis Blauwendraat, Andrew Singleton, Niccolo Mencacci, Katja Lohmann, Christine Klein, the Global Parkinson’s Genetic Program (GP2), Emilia M. Gatto, Marcelo Kauffman, Samson Khachatryan, Zaruhi Tavadyan, Claire E. Shepherd, Julie Hunter, Kishore Kumar, Melina Ellis, Miguel E. Rentería, Sulev Koks, Alexander Zimprich, Artur F. Schumacher-Schuh, Carlos Rieder, Paula Saffie Awad, Vitor Tumas, Sarah Camargos, Edward A. Fon, Oury Monchi, Ted Fon, Benjamin Pizarro Galleguillos, Marcelo Miranda, Maria Leonor Bustamante, Patricio Olguin, Pedro Chana, Beisha Tang, Huifang Shang, Jifeng Guo, Piu Chan, Wei Luo, Gonzalo Arboleda, Jorge Orozco, Marlene Jimenez del Rio, Alvaro Hernandez, Mohamed Salama, Walaa A. Kamel, Yared Z. Zewde, Alexis Brice, Jean-Christophe Corvol, Ana Westenberger, Anastasia Illarionova, Brit Mollenhauer, Christine Klein, Eva-Juliane Vollstedt, Franziska Hopfner, Günter Höglinger, Harutyun Madoev, Joanne Trinh, Johanna Junker, Katja Lohmann, Lara M. Lange, Manu Sharma, Sergio Groppa, Thomas Gasser, Zih-Hua Fang, Albert Akpalu, Georgia Xiromerisiou, Georgios Hadjigorgiou, Ioannis Dagklis, Ioannis Tarnanas, Leonidas Stefanis, Maria Stamelou, Efthymios Dadiotis, Alex Medina, Germaine Hiu-Fai Chan, Nancy Ip, Nelson Yuk-Fai Cheung, Phillip Chan, Xiaopu Zhou, Asha Kishore, Divya KP, Pramod Pal, Prashanth Lingappa Kukkle, Roopa Rajan, Rupam Borgohain, Mehri Salari, Andrea Quattrone, Enza Maria Valente, Lucilla Parnetti, Micol Avenali, Tommaso Schirinzi, Manabu Funayama, Nobutaka Hattori, Tomotaka Shiraishi, Altynay Karimova, Gulnaz Kaishibayeva, Cholpon Shambetova, Rejko Krüger, Ai Huey Tan, Azlina Ahmad-Annuar, Mohamed Ibrahim Norlinah, Nor Azian Abdul Murad, Norlinah Mohamed Ibrahim, Shahrul Azmin, Shen-Yang Lim, Wael Mohamed, Yi Wen Tay, Daniel Martinez-Ramirez, Mayela Rodriguez-Violante, Paula Reyes-Pérez, Bayasgalan Tserensodnom, Rajeev Ojha, Tim J. Anderson, Toni L. Pitcher, Arinola Sanyaolu, Njideka Okubadejo, Oluwadamilola Ojo, Jan O. Aasly, Lasse Pihlstrøm, Manuela Tan, Shoaib Ur-Rehman, Mario Cornejo-Olivas, Maria Leila Doquenia, Raymond Rosales, Angel Vinuela, Elena Iakovenko, Bashayer Al Mubarak, Muhammad Umair, Eng-King Tan, Jia Nee Foo, Ferzana Amod, Jonathan Carr, Soraya Bardien, Beomseok Jeon, Yun Joong Kim, Esther Cubo, Ignacio Alvarez, Janet Hoenicka, Katrin Beyer, Maria Teresa Periñan, Pau Pastor, Sarah El-Sadig, Christiane Zweier, Krack Paul, Chin-Hsien Lin, Hsiu-Chuan Wu, Pin-Jui Kung, Ruey-Meei Wu, Serena Wu, Yihru Wu, Rim Amouri, Samia Ben Sassi, A. Nazl Başak, Gencer Genc, Özgür Öztop Çakmak, Sibel Ertan, Alastair Noyce, Alejandro Martínez-Carrasco, Anette Schrag, Anthony Schapira, Camille Carroll, Claire Bale, Donald Grosset, Eleanor J. Stafford, Henry Houlden, Huw R. Morris, John Hardy, Kin Ying Mok, Mie Rizig, Nicholas Wood, Nigel Williams, Olaitan Okunoye, Patrick Alfryn Lewis, Rauan Kaiyrzhanov, Rimona Weil, Seth Love, Simon Stott, Simona Jasaitye, Sumit Dey, Vida Obese, Alberto Espay, Alyssa O’Grady, Andrew B. Singleton, Andrew K. Sobering, Bernadette Siddiqi, Bradford Casey, Brian Fiske, Cabell Jonas, Carlos Cruchaga, Caroline B. Pantazis, Charisse Comart, Claire Wegel, Cornelis Blauwendraat, Dan Vitale, Deborah Hall, Dena Hernandez, Ejaz Shiamim, Ekemini Riley, Faraz Faghri, Geidy E. Serrano, Hampton Leonard, Hirotaka Iwaki, Honglei Chen, Ignacio F. Mata, Ignacio Juan Keller Sarmiento, Jared Williamson, Jonggeol Jeff Kim, Joseph Jankovic, Joshua Shulman, Justin C. Solle, Kaileigh Murphy, Karen Nuytemans, Karl Kieburtz, Katerina Markopoulou, Kenneth Marek, Kristin S. Levine, Lana M. Chahine, Laurel Screven, Lauren Ruffrage, Lisa Shulman, Luca Marsili, Maggie Kuhl, Marissa Dean, Mary B. Makarious, Mathew Koretsky, Miguel Inca-Martinez, Mike A. Nalls, Naomi Louie, Niccolò Emanuele Mencacci, Roger Albin, Roy Alcalay, Ruth Walker, Sara Bandres-Ciga, Sohini Chowdhury, Sonya Dumanis, Steven Lubbe, Tao Xie, Tatiana Foroud, Thomas Beach, Todd Sherer, Yeajin Song, Duan Nguyen, Toan Nguyen, Masharip Atadzhanov

**Affiliations:** 1 Institute of Neurogenetics, University of Lübeck, Lübeck, Germany; 2 IRCCS Mondino Foundation, Pavia, Italy; 3 Department of Brain and Behavioral Sciences, University of Pavia, Pavia, Italy; 4 Northcott Neuroscience Laboratory, ANZAC Research Institute, Concord, NSW Australia; 5 Faculty of Medicine and Health, University of Sydney, Sydney, NSW Australia; 6 German Center for Neurodegenerative Diseases (DZNE), Tübingen, Germany; 7 Department of Neurology, Northwestern University Feinberg School of Medicine, Chicago, IL USA; 8 Division of Neurology, Department of Medicine, and the Mah Pooi Soo and Tan Chin Nam Centre for Parkinson’s and Related Disorders, Faculty of Medicine, University of Malaya, Kuala Lumpur, Malaysia; 9 Department of Molecular Medicine, University of Pavia, Pavia, Italy; 10 Department of Clinical Research, Michael J. Fox Foundation for Parkinson’s Research, New York City, NY USA; 11 Department of Medical and Molecular Genetics, Indiana University School of Medicine, Indianapolis, IN USA; 12 Garvan Institute of Medical Research, Darlinghurst, NSW Australia; 13 Molecular Medicine Laboratory and Neurology Department, Concord Repatriation General Hospital, The University of Sydney, Concord, NSW Australia; 14 Data Tecnica International, Washington, DC USA; 15 Center for Alzheimer’s and Related Dementias (CARD), National Institute on Aging and National Institute of Neurological Disorders and Stroke, National Institutes of Health, Bethesda, MD USA; 16 Molecular Genetics Section, Laboratory of Neurogenetics, National Institute on Aging, National Institutes of Health, Bethesda, MD USA; 17 Integrative Genomics Unit, Laboratory of Neurogenetics, National Institute on Aging, National Institutes of Health, Bethesda, MD USA; 18 Sanatorio de la Trinidad Mitre- INEBA, Buenos Aires, Argentina; 19 Hospital JM Ramos Mejia, Buenos Aires, Argentina; 20 Somnus Neurology Clinic, Yerevan, Armenia; 21 Neuroscience Research Australia, Sydney, NSW Australia; 22 ANZAC Research Institute, Concord, NSW Australia; 23 Garvan Institute of Medical Research and Concord Repatriation General Hospital, Darlinghurst, NSW Australia; 24 Concord Hospital, Concord, NSW Australia; 25 QIMR Berghofer Medical Research Institute, Herston, QL Australia; 26 Murdoch University, Perth, Australia; 27 Medical University Vienna Austria, Vienna, Austria; 28 Universidade Federal do Rio Grande do Sul / Hospital de Clínicas de Porto Alegre, Porto Alegre, Brazil; 29 Federal University of Health Sciences of Porto Alegre, Porto Alegre, Brazil; 30 Universidade Federal do Rio Grande do Sul, Porto Alegre, Brazil; 31 University of São Paulo, São Paulo, Brazil; 32 Universidade Federal de Minas Gerais, Belo Horizonte, Brazil; 33 Montreal Neurological Institute, Montreal, QC Canada; 34 Institut universitaire de gériatrie de Montréal, Montreal, QC Canada; 35 McGill University, Montreal, QC Canada; 36 Universidad de Chile, Santiago, Chile; 37 Fundación Diagnosis, Santiago, Chile; 38 Faculty of Medicine Universidad de Chile, Santiago, Chile; 39 CETRAM, Santiago, Chile; 40 Central South University, Changsha, China; 41 West China Hospital Sichuan University, Chengdu, China; 42 Xiangya Hospital, Changsha, China; 43 Capital Medical University, Beijing, China; 44 Zhejiang University, Hangzhou, China; 45 Universidad Nacional de Colombia, Bogotá, Colombia; 46 Fundación Valle del Lili, Santiago De Cali, Colombia; 47 University of Antioquia, Medellin, Colombia; 48 University of Costa Rica, San Jose, Costa Rica; 49 The American University in Cairo, Cairo, Egypt; 50 Beni-Suef University, Beni Suef, Egypt; 51 Addis Ababa University, Addis Ababa, Ethiopia; 52 Paris Brain Institute, Paris, France; 53 Sorbonne Université, Paris, France; 54 University of Lübeck, Lübeck, Germany; 55 Deutsches Zentrum für Neurodegenerative Erkrankungen, Göttingen, Germany; 56 University Medical Center Göttingen, Göttingen, Germany; 57 University Hospital, LMU Munich, Munich, Germany; 58 Hannover Medical School, Hannover, Germany; 59 University of Lübeck and University Medical Center Schleswig-Holstein, Lübeck, Germany; 60 University of Tubingen, Tübingen, Germany; 61 University of Mainz, Mainz, Germany; 62 The German Center for Neurodegenerative Diseases, Göttingen, Germany; 63 University of Ghana Medical School, Accra, Ghana; 64 University of Thessaly, Volos, Greece; 65 Aristotle University of Thessaloniki, Thessaloniki, Greece; 66 Ionian University, Corfu, Greece; 67 Biomedical research Foundation of the Academy of Athens, Athens, Greece; 68 Diagnostic and Therapeutic Centre HYGEIA Hospital, Marousi, Greece; 69 Hospital San Felipe, Tegucigalpa, Honduras; 70 Queen Elizabeth Hospital, Kowloon, Hong Kong; 71 The Hong Kong University of Science and Technology, Kowloon, Hong Kong; 72 Aster Medcity, Kochi, India; 73 Sree Chitra Tirunal Institute for Medical Sciences and Technology, Thiruvananthapuram, India; 74 National Institute of Mental Health & Neurosciences, Bengaluru, India; 75 Manipal Hospital, Delhi, India; 76 All India Institute of Medical Sciences, Delhi, India; 77 Nizam’s Institute Of Medical Sciences, Hyderabad, India; 78 Shahid Beheshti University of Medical Science, Tehran, Iran; 79 Magna Græcia University of Catanzaro, Catanzaro, Italy; 80 University of Pavia, Pavia, Italy; 81 University of Perugia, Perugia, Italy; 82 University of Rome Tor Vergata, Rome, Italy; 83 Juntendo University, Tokyo, Japan; 84 Juntendo University faculty of medicine, Tokyo, Japan; 85 Jikei University School of Medicine, Tokyo, Japan; 86 Institute of Neurology and Neurorehabilitation, Almaty, Kazakhstan; 87 Kyrgyz State Medical Academy, Bishkek, Kyrgyzstan; 88 University of Luxembourg, Luxembourg, Luxembourg; 89 University of Malaya, Kuala Lumpur, Malaysia; 90 Universiti Kebangsaan Malaysia, Selangor, Malaysia; 91 UKM Medical Molecular Biology Institute, Kuala Lumpur, Malaysia; 92 Universiti Kebangsaan Malaysia Medical Centre, Kuala Lumpur, Malaysia; 93 International Islamic University, Kuala Lumpur, Malaysia; 94 Tecnologico de Monterrey, Monterrey, Mexico; 95 Instituto Nacional de Neurologia y Neurocirugia, Mexico City, Mexico; 96 Universidad Nacional Autónoma de México, Mexico City, Mexico; 97 Mongolian National University of Medical Sciences, Ulaanbaatar, Mongolia; 98 Tribhuvan University, Kirtipur, Nepal; 99 University of Otago, Dunedin, New Zealand; 100 University of Lagos, Lagos, Nigeria; 101 College of Medicine of the University of Lagos, Lagos, Nigeria; 102 Norwegian University of Science and Technology, Trondheim, Norway; 103 Oslo University Hospital, Oslo, Norway; 104 University of Science and Technology Bannu, Bannu, Pakistan; 105 Instituto Nacional de Ciencias Neurologicas, Lima, Peru; 106 Metropolitan Medical Center, Manila, Philippines; 107 University of Puerto Rico, San Juan, Puerto Rico; 108 Research Center of Neurology, Moscow, Russia; 109 King Faisal Specialist Hospital and Research Center, Riyadh, Saudi Arabia; 110 King Abdullah International Medical Research Center, Jeddah, Saudi Arabia; 111 National Neuroscience Institute, Singapore, Singapore; 112 Nanyang Technological University, Singapore, Singapore; 113 University of KwaZulu-Natal, Durban, South Africa; 114 University of Stellenbosch, Stellenbosch, South Africa; 115 Stellenbosch University, Stellenbosch, South Africa; 116 Seoul National University Hospital, Seoul, South Korea; 117 Yongin Severance Hospital, Seoul, South Korea; 118 Hospital Universitario Burgos, Burgos, Spain; 119 University Hospital Mutua Terrassa, Barcelona, Spain; 120 Institut de Recerca Sant Joan de Deu, Barcelona, Spain; 121 Research Institute Germans Trias i Pujol, Barcelona, Spain; 122 Instituto de Biomedicina de Sevilla, Seville, Spain; 123 University Hospital Germans Trias i Pujol, Barcelona, Spain; 124 Faculty of medicine university of Khartoum, Khartoum, Sudan; 125 Inselspital Bern, University of Bern, Bern, Switzerland; 126 National Taiwan University Hospital, Taipei City, Taiwan; 127 Chang Gung Memorial Hospital, Taoyuan City, Taiwan; 128 National Taiwan University, Taipei City, Taiwan; 129 Chang Gung University, Taoyuan City, Taiwan; 130 National Institute Mongi Ben Hamida of Neurology, Tunis, Tunisia; 131 Mongi Ben Hmida National Institute of Neurology, Tunis, Tunisia; 132 Koç University, Istanbul, Turkey; 133 Sisli Etfal Training and Research Hospital, Istanbul, Turkey; 134 Queen Mary University of London, London, United Kingdom; 135 University College London, London, United Kingdom; 136 University of Plymouth, Plymouth, United Kingdom; 137 Parkinson’s UK, London, United Kingdom; 138 University of Glasgow, Glasgow, United Kingdom; 139 Univeristy College London, London, United Kingdom; 140 Cardiff University, Cardiff, United Kingdom; 141 Royal Veterinary College University of London, London, United Kingdom; 142 University of Bristol, Bristol, United Kingdom; 143 Cure Parkinson’s, London, United Kingdom; 144 University of Cincinnati, Cincinnati, OH USA; 145 The Michael J. Fox Foundation for Parkinson’s Research, New York, NY USA; 146 National Institute on Aging, Bethesda, MD USA; 147 Augusta University / University of Georgia Medical Partnership, Augusta, GA USA; 148 Mid-Atlantic Permanente Medical Group, Bethesda, MD USA; 149 Washington University, St. Louis, MO USA; 150 National Institutes of Health, Bethesda, MD USA; 151 Indiana University, Bloomington, IN USA; 152 Rush University, Chicago, IL USA; 153 Kaiser Permanente, Oakland, CA USA; 154 Coalition for Aligning Science, Washington, WA USA; 155 Banner Sun Health Research Institute, Sun City, AZ USA; 156 National Institute on Aging/National Institutes of Health, Bethesda, MD USA; 157 Data Tecnica International, Washington, WA USA; 158 Michigan State University, East Lansing, MI USA; 159 Cleveland Clinic, Cleveland, OH USA; 160 Northwestern University, Evanston, IL USA; 161 Baylor College of Medicine, Houston, TX USA; 162 Baylor College of Medicine / Texas Children’s Hospital, Houston, TX USA; 163 University of Miami Miller School of Medicine, Miami, FL USA; 164 Beth Israel Deaconess Medical Center, Boston, MA USA; 165 North Shore University Health System, Chicago, IL USA; 166 Institute for Neurodegenerative Disorders, New Haven, CT USA; 167 University of Pittsburgh, Pittsburgh, PA USA; 168 University of Alabama at Birmingham, Birmingham, AL USA; 169 University of Maryland, Baltimore, MD USA; 170 Universit of Michigan, Ann Arbor, MI USA; 171 Columbia University, New York, NY USA; 172 James J. Peters Veterans Affairs Medical Center, New York, NY USA; 173 Aligning Science Across Parkinson’s, Washington, WA USA; 174 University of Chicago, Chicago, IL USA; 175 Indiana University School of Medicine, Indianapolis, IN USA; 176 Sun Health Research Institution, Sun City, AZ USA; 177 Hue University, Huế, Vietnam; 178 University of Zambia, Lusaka, Zambia

**Keywords:** Sequencing, Parkinson's disease

Correction to: *npj Parkinson’s Disease* 10.1038/s41531-023-00526-9, published online 27 June 2023

In this article the Global Parkinson’s Genetics Program (GP2) members names and affiliations were missing in the main author list of the Original article which are listed in the below.

Emilia M. Gatto^18^, Marcelo Kauffman^19^, Samson Khachatryan^20^, Zaruhi Tavadyan^20^, Claire E. Shepherd^21^, Julie Hunter^22^, Kishore Kumar^23^, Melina Ellis^24^, Miguel E. Rentería^25^, Sulev Koks^26^, Alexander Zimprich^27^, Artur F. Schumacher-Schuh^28^, Carlos Rieder^29^, Paula Saffie Awad^30^, Vitor Tumas^31^, Sarah Camargos^32^, Edward A. Fon^33^, Oury Monchi^34^, Ted Fon^35^, Benjamin Pizarro Galleguillos^36^, Marcelo Miranda^37^, Maria Leonor Bustamante^38^, Patricio Olguin^36^, Pedro Chana^39^, Beisha Tang^40^, Huifang Shang^41^, Jifeng Guo^42^, Piu Chan^43^, Wei Luo^44^, Gonzalo Arboleda^45^, Jorge Orozco^46^, Marlene Jimenez del Rio^47^, Alvaro Hernandez^48^, Mohamed Salama^49^, Walaa A. Kamel^50^, Yared Z. Zewde^51^, Alexis Brice^52^, Jean-Christophe Corvol^53^, Ana Westenberger^54^, Anastasia Illarionova^55^, Brit Mollenhauer^56^, Christine Klein^54^, Eva-Juliane Vollstedt^54^, Franziska Hopfner^57^, Günter Höglinger^58^, Harutyun Madoev^54^, Joanne Trinh^54^, Johanna Junker^54^, Katja Lohmann^54^, Lara M. Lange^59^, Manu Sharma^60^, Sergio Groppa^61^, Thomas Gasser^60^, Zih-Hua Fang^62^, Albert Akpalu^63^, Georgia Xiromerisiou^64^, Georgios Hadjigorgiou^64^, Ioannis Dagklis^65^, Ioannis Tarnanas^66^, Leonidas Stefanis^67^, Maria Stamelou^68^, Efthymios Dadiotis^64^, Alex Medina^69^, Germaine Hiu-Fai Chan^70^, Nancy Ip^71^, Nelson Yuk-Fai Cheung^70^, Phillip Chan^71^, Xiaopu Zhou^71^, Asha Kishore^72^, Divya KP^73^, Pramod Pal^74^, Prashanth Lingappa Kukkle^75^, Roopa Rajan^76^, Rupam Borgohain^77^, Mehri Salari^78^, Andrea Quattrone^79^, Enza Maria Valente^80^, Lucilla Parnetti^81^, Micol Avenali^80^, Tommaso Schirinzi^82^, Manabu Funayama^83^, Nobutaka Hattori^84^, Tomotaka Shiraishi^85^, Altynay Karimova^86^, Gulnaz Kaishibayeva^86^, Cholpon Shambetova^87^, Rejko Krüger^88^, Ai Huey Tan^89^, Azlina Ahmad-Annuar^89^, Mohamed Ibrahim Norlinah^90^, Nor Azian Abdul Murad^91^, Norlinah Mohamed Ibrahim^92^, Shahrul Azmin^92^, Shen-Yang Lim^89^, Wael Mohamed^93^, Yi Wen Tay^89^, Daniel Martinez-Ramirez^94^, Mayela Rodriguez-Violante^95^, Paula Reyes-Pérez^96^, Bayasgalan Tserensodnom^97^, Rajeev Ojha^98^, Tim J. Anderson^99^, Toni L. Pitcher^99^, Arinola Sanyaolu^100^, Njideka Okubadejo^100^, Oluwadamilola Ojo^101^, Jan O. Aasly^102^, Lasse Pihlstrøm^103^, Manuela Tan^103^, Shoaib Ur-Rehman^104^, Mario Cornejo-Olivas^105^, Maria Leila Doquenia^106^, Raymond Rosales^106^, Angel Vinuela^107^, Elena Iakovenko^108^, Bashayer Al Mubarak^109^, Muhammad Umair^110^, Eng-King Tan^111^, Jia Nee Foo^112^, Ferzana Amod^113^, Jonathan Carr^114^, Soraya Bardien^115^, Beomseok Jeon^116^, Yun Joong Kim^117^, Esther Cubo^118^, Ignacio Alvarez^119^, Janet Hoenicka^120^, Katrin Beyer^121^, Maria Teresa Periñan^122^, Pau Pastor^123^, Sarah El-Sadig^124^, Christiane Zweier^125^, Krack Paul^125^, Chin-Hsien Lin^126^, Hsiu-Chuan Wu^127^, Pin-Jui Kung^128^, Ruey-Meei Wu^126^, Serena Wu^129^, Yihru Wu^127^, Rim Amouri^130^, Samia Ben Sassi^131^, A. Nazl Başak^132^, Gencer Genc^133^, Özgür Öztop Çakmak^132^, Sibel Ertan^132^, Alastair Noyce^134^, Alejandro Martínez-Carrasco^135^, Anette Schrag^135^, Anthony Schapira^135^, Camille Carroll^136^, Claire Bale^137^, Donald Grosset^138^, Eleanor J. Stafford^135^, Henry Houlden^135^, Huw R. Morris^135^, John Hardy^135^, Kin Ying Mok^139^, Mie Rizig^135^, Nicholas Wood^135^, Nigel Williams^140^, Olaitan Okunoye^135^, Patrick Alfryn Lewis^141^, Rauan Kaiyrzhanov^135^, Rimona Weil^135^, Seth Love^142^, Simon Stott^143^, Simona Jasaitye^135^, Sumit Dey^134^, Vida Obese^135^, Alberto Espay^144^, Alyssa O’Grady^145^, Andrew B. Singleton^146^, Andrew K. Sobering^147^, Bernadette Siddiqi^145^, Bradford Casey^145^, Brian Fiske^145^, Cabell Jonas^148^, Carlos Cruchaga^149^, Caroline B. Pantazis^150^, Charisse Comart^145^, Claire Wegel^151^, Cornelis Blauwendraat^150^, Dan Vitale^150^, Deborah Hall^152^, Dena Hernandez^150^, Ejaz Shiamim^153^, Ekemini Riley^154^, Faraz Faghri^150^, Geidy E. Serrano^155^, Hampton Leonard^156^, Hirotaka Iwaki^157^, Honglei Chen^158^, Ignacio F. Mata^159^, Ignacio Juan Keller Sarmiento^160^, Jared Williamson^153^, Jonggeol Jeff Kim^150^, Joseph Jankovic^161^, Joshua Shulman^162^, Justin C. Solle^145^, Kaileigh Murphy^145^, Karen Nuytemans^163^, Karl Kieburtz^164^, Katerina Markopoulou^165^, Kenneth Marek^166^, Kristin S. Levine^157^, Lana M. Chahine^167^, Laurel Screven^146^, Lauren Ruffrage^168^, Lisa Shulman^169^, Luca Marsili^144^, Maggie Kuhl^145^, Marissa Dean^168^, Mary B. Makarious^150^, Mathew Koretsky^150^, Miguel Inca-Martinez^159^, Mike A. Nalls^150^, Naomi Louie^145^, Niccolò Emanuele Mencacci^160^, Roger Albin^170^, Roy Alcalay^171^, Ruth Walker^172^, Sara Bandres-Ciga^150^, Sohini Chowdhury^145^, Sonya Dumanis^173^, Steven Lubbe^160^, Tao Xie^174^, Tatiana Foroud^175^, Thomas Beach^176^, Todd Sherer^145^, Yeajin Song^150^, Duan Nguyen^177^, Toan Nguyen^177^, Masharip Atadzhanov^178^.

^18^Sanatorio de la Trinidad Mitre- INEBA, Buenos Aires, Argentina, ^19^Hospital JM Ramos Mejia, Buenos Aires, Argentina, ^20^Somnus Neurology Clinic, Yerevan, Armenia, ^21^Neuroscience Research Australia, Sydney, NSW, Australia, ^22^ANZAC Research Institute, Concord, NSW, Australia, ^23^Garvan Institute of Medical Research and Concord Repatriation General Hospital, Darlinghurst, NSW, Australia, ^24^Concord Hospital, Concord, NSW, Australia, ^25^QIMR Berghofer Medical Research Institute, Herston, QL, Australia, ^26^Murdoch University, Perth, Australia, ^27^Medical University Vienna Austria, Vienna, Austria, ^28^Universidade Federal do Rio Grande do Sul / Hospital de Clínicas de Porto Alegre, Porto Alegre, Brazil, ^29^Federal University of Health Sciences of Porto Alegre, Porto Alegre, Brazil, ^30^Universidade Federal do Rio Grande do Sul, Porto Alegre, Brazil, ^31^University of São Paulo, São Paulo, Brazil, ^32^Universidade Federal de Minas Gerais, Belo Horizonte, Brazil, ^33^Montreal Neurological Institute, Montreal, QC, Canada, ^34^Institut universitaire de gériatrie de Montréal, Montreal, QC, Canada, ^35^McGill University, Montreal, QC, Canada, ^36^Universidad de Chile, Santiago, Chile, ^37^Fundación Diagnosis, Santiago, Chile, ^38^Faculty of Medicine Universidad de Chile, Santiago, Chile, ^39^CETRAM, Santiago, Chile, ^40^Central South University, Changsha, China, ^41^West China Hospital Sichuan University, Chengdu, China, ^42^Xiangya Hospital, Changsha, China, ^43^Capital Medical University, Beijing, China, ^44^Zhejiang University, Hangzhou, China, ^45^Universidad Nacional de Colombia, Bogotá, Colombia, ^46^Fundación Valle del Lili, Santiago De Cali, Colombia, ^47^University of Antioquia, Medellin, Colombia, ^48^University of Costa Rica, San Jose, Costa Rica, ^49^The American University in Cairo, Cairo, Egypt, ^50^Beni-Suef University, Beni Suef, Egypt, ^51^Addis Ababa University, Addis Ababa, Ethiopia, ^52^Paris Brain Institute, Paris, France, ^53^Sorbonne Université, Paris, France, ^54^University of Lübeck, Lübeck, Germany, ^55^Deutsches Zentrum für Neurodegenerative Erkrankungen, Göttingen, Germany, ^56^University Medical Center Göttingen, Göttingen, Germany, ^57^University Hospital, LMU Munich, Munich, Germany, ^58^Hannover Medical School, Hannover, Germany, ^59^University of Lübeck and University Medical Center Schleswig-Holstein, Lübeck, Germany, ^60^University of Tubingen, Tübingen, Germany, ^61^University of Mainz, Mainz, Germany, ^62^The German Center for Neurodegenerative Diseases, Göttingen, Germany, ^63^University of Ghana Medical School, Accra, Ghana, ^64^University of Thessaly, Volos, Greece, ^65^Aristotle University of Thessaloniki, Thessaloniki, Greece, ^66^Ionian University, Corfu, Greece, ^67^Biomedical research Foundation of the Academy of Athens, Athens, Greece, ^68^Diagnostic and Therapeutic Centre HYGEIA Hospital, Marousi, Greece, ^69^Hospital San Felipe, Tegucigalpa, Honduras, ^70^Queen Elizabeth Hospital, Kowloon, Hong Kong, ^71^The Hong Kong University of Science and Technology, Kowloon, Hong Kong, ^72^Aster Medcity, Kochi, India, ^73^Sree Chitra Tirunal Institute for Medical Sciences and Technology, Thiruvananthapuram, India, ^74^National Institute of Mental Health & Neurosciences, Bengaluru, India, ^75^Manipal Hospital, Delhi, India, ^76^All India Institute of Medical Sciences, Delhi, India, ^77^Nizam’s Institute Of Medical Sciences, Hyderabad, India, ^78^Shahid Beheshti University of Medical Science, Tehran, Iran, ^79^Magna Græcia University of Catanzaro, Catanzaro, Italy, ^80^University of Pavia, Pavia, Italy, ^81^University of Perugia, Perugia, Italy, ^82^University of Rome Tor Vergata, Rome, Italy, ^83^Juntendo University, Tokyo, Japan, ^84^Juntendo University faculty of medicine, Tokyo, Japan, ^85^Jikei University School of Medicine, Tokyo, Japan, ^86^Institute of Neurology and Neurorehabilitation, Almaty, Kazakhstan, ^87^Kyrgyz State Medical Academy, Bishkek, Kyrgyzstan, ^88^University of Luxembourg, Luxembourg, Luxembourg, ^89^University of Malaya, Kuala Lumpur, Malaysia, ^90^Universiti Kebangsaan Malaysia, Selangor, Malaysia, ^91^UKM Medical Molecular Biology Institute, Kuala Lumpur, Malaysia, ^92^Universiti Kebangsaan Malaysia Medical Centre, Kuala Lumpur, Malaysia, ^93^International Islamic University, Kuala Lumpur, Malaysia, ^94^Tecnologico de Monterrey, Monterrey, Mexico, ^95^Instituto Nacional de Neurologia y Neurocirugia, Mexico City, Mexico, ^96^Universidad Nacional Autónoma de México, Mexico City, Mexico, ^97^Mongolian National University of Medical Sciences, Ulaanbaatar, Mongolia, ^98^Tribhuvan University, Kirtipur, Nepal, ^99^University of Otago, Dunedin, New Zealand, ^100^University of Lagos, Lagos, Nigeria, ^101^College of Medicine of the University of Lagos, Lagos, Nigeria, ^102^Norwegian University of Science and Technology, Trondheim, Norway, ^103^Oslo University Hospital, Oslo, Norway, ^104^University of Science and Technology Bannu, Bannu, Pakistan, ^105^Instituto Nacional de Ciencias Neurologicas, Lima, Peru, ^106^Metropolitan Medical Center, Manila, Philippines, ^107^University of Puerto Rico, San Juan, Puerto Rico, ^108^Research Center of Neurology, Moscow, Russia, ^109^King Faisal Specialist Hospital and Research Center, Riyadh, Saudi Arabia, ^110^King Abdullah International Medical Research Center, Jeddah, Saudi Arabia, ^111^National Neuroscience Institute, Singapore, Singapore, ^112^Nanyang Technological University, Singapore, Singapore, ^113^University of KwaZulu-Natal, Durban, South Africa, ^114^University of Stellenbosch, Stellenbosch, South Africa, ^115^Stellenbosch University, Stellenbosch, South Africa, ^116^Seoul National University Hospital, Seoul, South Korea, ^117^Yongin Severance Hospital, Seoul, South Korea, ^118^Hospital Universitario Burgos, Burgos, Spain, ^119^University Hospital Mutua Terrassa, Barcelona, Spain, ^120^Institut de Recerca Sant Joan de Deu, Barcelona, Spain, ^121^Research Institute Germans Trias i Pujol, Barcelona, Spain, ^122^Instituto de Biomedicina de Sevilla, Seville, Spain, ^123^University Hospital Germans Trias i Pujol, Barcelona, Spain, ^124^Faculty of medicine university of Khartoum, Khartoum, Sudan, ^125^Inselspital Bern, University of Bern, Bern, Switzerland, ^126^National Taiwan University Hospital, Taipei City, Taiwan, ^127^Chang Gung Memorial Hospital, Taoyuan City, Taiwan, ^128^National Taiwan University, Taipei City, Taiwan, ^129^Chang Gung University, Taoyuan City, Taiwan, ^130^National Institute Mongi Ben Hamida of Neurology, Tunis, Tunisia, ^131^Mongi Ben Hmida National Institute of Neurology, Tunis, Tunisia, ^132^Koç University, Istanbul, Turkey, ^133^Sisli Etfal Training and Research Hospital, Istanbul, Turkey, ^134^Queen Mary University of London, London, United Kingdom, ^135^University College London, London, United Kingdom, ^136^University of Plymouth, Plymouth, United Kingdom, ^137^Parkinson’s UK, London, United Kingdom, ^138^University of Glasgow, Glasgow, United Kingdom, ^139^Univeristy College London, London, United Kingdom, ^140^Cardiff University, Cardiff, United Kingdom, ^141^Royal Veterinary College University of London, London, United Kingdom, ^142^University of Bristol, Bristol, United Kingdom, ^143^Cure Parkinson’s, London, United Kingdom, ^144^University of Cincinnati, Cincinnati, OH, USA, ^145^The Michael J. Fox Foundation for Parkinson’s Research, New York, NY, USA, ^146^National Institute on Aging, Bethesda, MD, USA, ^147^Augusta University / University of Georgia Medical Partnership, Augusta, GA, USA, ^148^Mid-Atlantic Permanente Medical Group, Bethesda, MD, USA, ^149^Washington University, St. Louis, MO, USA, ^150^National Institutes of Health, Bethesda, MD, USA, ^151^Indiana University, Bloomington, IN, USA, ^152^Rush University, Chicago, IL, USA, ^153^Kaiser Permanente, Oakland, CA, USA, ^154^Coalition for Aligning Science, Washington, WA, USA, ^155^Banner Sun Health Research Institute, Sun City, AZ, USA, ^156^National Institute on Aging/National Institutes of Health, Bethesda, MD, USA, ^157^Data Tecnica International, Washington, WA, USA, ^158^Michigan State University, East Lansing, MI, USA, ^159^Cleveland Clinic, Cleveland, OH, USA, ^160^Northwestern University, Evanston, IL, USA, ^161^Baylor College of Medicine, Houston, TX, USA, ^162^Baylor College of Medicine / Texas Children’s Hospital, Houston, TX, USA, ^163^University of Miami Miller School of Medicine, Miami, FL, USA, ^164^Beth Israel Deaconess Medical Center, Boston, MA, USA, ^165^North Shore University Health System, Chicago, IL, USA, ^166^Institute for Neurodegenerative Disorders, New Haven, CT, USA, ^167^University of Pittsburgh, Pittsburgh, PA, USA, ^168^University of Alabama at Birmingham, Birmingham, AL, USA, ^169^University of Maryland, Baltimore, MD, USA, ^170^Universit of Michigan, Ann Arbor, MI, USA, ^171^Columbia University, New York, NY, USA, ^172^James J. Peters Veterans Affairs Medical Center, New York, NY, USA, ^173^Aligning Science Across Parkinson’s, Washington, WA, USA, ^174^University of Chicago, Chicago, IL, USA, ^175^Indiana University School of Medicine, Indianapolis, IN, USA, ^176^Sun Health Research Institution, Sun City, AZ, USA, ^177^Hue University, Huế, Vietnam, ^178^University of Zambia, Lusaka, Zambia.

