# Supplementary material for: Elucidating causative gene variants in hereditary Parkinson’s disease in the Global Parkinson’s Genetics Program (GP2)
Source: NPJ Parkinsons Dis. 2023 Jun 27;9:100. doi: 10.1038/s41531-023-00526-9 (PMC10300084; doi:10.1038/s41531-023-00526-9)
Supplement: Supplementary file 1 — Supplementary Material [file 41531_2023_526_MOESM1_ESM.pdf]

Supplementary material

Elucidating causative gene variants in hereditary Parkinson's disease in the Global Parkinson's Genetics Program (GP2)

Lara M. Lange, MD,[1] Micol Avenali, MD,[2,3] Melina Ellis, BSc,[4] Anastasia Illarionova,[5] Ignacio J. Keller Sarmiento, MD,[6] Ai-Huey Tan, MD, PhD, FRCP,[7] Harutyun Madoev, MS,[1] Caterina Galandra,[2,8] Johanna Junker, MD,[1] Karisha Roopnarain, MD,[1] Justin Solle,[9] Claire Wegel,[10] Zih-Hua Fang, PhD,[5] Peter Heutink, PhD,[5] Kishore R. Kumar, MBBS, PhD, FRACP,[11] Shen-Yang Lim, MD, FRACP,[7] Enza Maria Valente, MD,[2,8] Mike Nalls,[12,14,15] Cornelis Blauwendraat,[13,14] Andrew Singleton,[14,15] Niccolo Mencacci, MD, PhD,[6] Katja Lohmann, PhD,[1] Christine Klein, MD,[1,*] *and the Global Parkinson's Genetic Program (GP2)*

[1] *Institute of Neurogenetics, University of Lübeck, Lübeck, Germany*
[2] *IRCCS Mondino Foundation, Pavia, Italy*
[3] *Department of Brain and Behavioral Sciences, University of Pavia, Pavia, Italy*
[4] *Northcott Neuroscience Laboratory, ANZAC Research Institute, Concord, New South Wales, Australia; Faculty of Medicine and Health, University of Sydney, Sydney, NSW, Australia.*
[5] *German Center for Neurodegenerative Diseases (DZNE), Tübingen, Germany*
[6] *Department of Neurology, Northwestern University Feinberg School of Medicine, Chicago, IL, USA*
[7] *Division of Neurology, Department of Medicine, and the Mah Pooi Soo and Tan Chin Nam Centre for Parkinson's and Related Disorders, Faculty of Medicine, University of Malaya, Kuala Lumpur, Malaysia*
[8] *Department of Molecular Medicine, University of Pavia, Pavia, Italy*
[9] *Department of Clinical Research, Michael J. Fox Foundation for Parkinson's Research, New York City, NY, USA*
[10] *Department of Medical and Molecular Genetics, Indiana University School of Medicine, Indianapolis, IN, USA*
[11] *Garvan Institute of Medical Research, Darlinghurst, New South Wales, Australia; Molecular Medicine Laboratory and Neurology Department, Concord Repatriation General Hospital, The University of Sydney, Concord, New South Wales, Australia.*
[12] *Data Tecnica International, Washington, DC, USA*
[13] *Integrative Genomics Unit, Laboratory of Neurogenetics, National Institute on Aging, National Institutes of Health, Bethesda, MD, USA*
[14] *Center for Alzheimer's and Related Dementias (CARD), National Institute on Aging and National Institute of Neurological Disorders and Stroke, National Institutes of Health, Bethesda, MD, USA*
[15] *Molecular Genetics Section, Laboratory of Neurogenetics, National Institute on Aging, National Institutes of Health, Bethesda, MD, USA*

Supplementary Table 1. Sample prioritization criteria.

	Family history/ Available samples	Age at onset (AAO)	Ethnicity
**Very strong**	> 2 affected	<20 years	Both parents with non-European ancestry (underrepresented)*
**Strong**	1 affected, both healthy parents	21-40 years	One side/parent with non-European ancestry (underrepresented)*
**Medium**	2 affected	41-50 years	Other non-European
**Possibly**	1 affected but strong positive family history	>50 years	White/European

*: Africa, Latin America/Caribbean, Indigenous Americans/Oceania, Middle East, and non-East Asians

This table lists the criteria the Monogenic Network uses to evaluate the likelihood of a monogenic cause of the disease. Based on these criteria listed in the table as well as the presence of consanguinity, submitted samples will be prioritized for WGS

Global Parkinson's Genetics Program (GP2) Banner Authors

This project was supported by the Global Parkinson's Genetics Program (GP2). GP2 is funded by the Aligning Science Against Parkinson's (ASAP) initiative and implemented by The Michael J. Fox Foundation for Parkinson's Research (**https://gp2.org**)

Members: 206
Version date: November 10, 2022

Country	Name	Institution	Funders and Disclosures
Argentina	Emilia M Gatto	Sanatorio de la Trinidad Mitre - INEBA	Nothing to declare
	Marcelo Kauffman	Hospital JM Ramos Mejia	I am an employee of CONICET
Armenia	Samson Khachatryan	Somnus Neurology Clinic	Nothing to declare
	Zaruhi Tavadyan	Somnus Neurology Clinic	Nothing to declare
Australia	Claire E Shepherd	Neuroscience Research Australia	The Sydney Brain Bank is located at and supported by Neuroscience Research Australia
	Julie Hunter	ANZAC Research Institute	Nothing to declare
	Kishore Kumar	Garvan Institute of Medical Research and Concord Repatriation General Hospital	Paul Ainsworth Family Foundation
	Melina Ellis	Concord Hospital	Nothing to declare
	Miguel E. Rentería	QIMR Berghofer Medical Research Institute	The Australian Parkinson's Genetics Study is supported by the Shake It Up Australia Foundation and The Michael J. Fox Foundation for Parkinson's Research
	Sulev Koks	Murdoch University	Nothing to declare
Austria	Alexander Zimprich	Medical University Vienna Austria	Nothing to declare
Brazil	Artur F. Schumacher-Schuh	Universidade Federal do Rio Grande do Sul / Hospital de Clínicas de Porto Alegre	Nothing to declare

	Carlos Rieder	Federal University of Health Sciences of Porto Alegre	Nothing to declare
	Vitor Tumas	University of São Paulo	Nothing to declare
Canada	Edward A. Fon	Montreal Neurological Institute	Nothing to declare
	Oury Monchi	Institut universitaire de gériatrie de Montréal	CIHR, Brain Canada, Parkinson Canada
	Ted Fon	McGill University	Nothing to declare
Chile	Benjamin Pizarro	Universidad de Chile	Nothing to declare
	Maria Leonor Bustamante	Faculty of Medicine Universidad de Chile	Nothing to declare
	Marcelo Miranda	Fundación Diagnosis	Nothing to declare
	Patricio Olguin	University of Chile	Nothing to declare
	Pedro Chana	CETRAM	Nothing to declare
China	Beisha Tang	Central South University	Nothing to declare
	Huifang Shang	West China Hospital Sichuan University	Nothing to declare
	Jifeng Guo	Xiangya Hospital	Nothing to declare
	Piu Chan	Capital Medical University	Nothing to declare
	Wei Luo	Zhejiang University	Nothing to declare
	Xiaopu Zhou	The Hong Kong University of Science and Technology	Nothing to declare
Colombia	Gonzalo Arboleda	Universidad Nacional de Colombia	Nothing to declare
	Jorge Orozco	Fundación Valle del Lili	Nothing to declare
	Marlene Jimenez del Rio	University of Antioquia	Nothing to declare
Costa Rica	Alvaro Hernandez	University of Costa Rica	Nothing to declare
Egypt	Mohamed Salama	The American University in Cairo	The AUC/ ASRT/ DAAD
	Walaa A. Kamel	Beni-Suef University	Nothing to declare
France	Alexis Brice	Paris Brain Institute	Nothing to declare
	Jean-Christophe Corvol	Sorbonne Université	Nothing to declare
Germany	Anastasia Illarionova	Deutsches Zentrum für Neurodegenerative Erkrankungen	Nothing to declare

	Brit Mollenhauer	University Medical Center Göttingen	Nothing to declare
	Christine Klein	University of Luebeck	CK serves as a medical Advisor to Centogene on genetic testing reports in the field of movement disorders, except Parkinson's disease, and is a member of the Scientific Advisory Board of Retromer Therapeutics
	Eva-Juliane Vollstedt	University of Luebeck	Nothing to declare
	Günter Höglinger	Hannover Medical School	Nothing to declare
	Harutyun Madoev	University of Luebeck	Nothing to declare
	Johanna Junker	University of Luebeck	Nothing to declare
	Karisha Roopnarain	University of Luebeck	Nothing to declare
	Katja Lohmann	University of Luebeck	Nothing to declare
	Lara M. Lange	University of Lübeck and University Medical Center Schleswig-Holstein	Nothing to declare
	Manu Sharma	University of Tubingen	Dr. Sharma is further funded by the Michael J Fox Foundation, USA Genetic Diversity in PD Program: GAP-India Grant ID: 009411.
	Peter Heutink	German Center for Neurodegenerative Diseases (DZNE)	Consultant for Neuron23 Inc.
	Sergio Groppa	University of Mainz	Nothing to declare
	Thomas Gasser	University of Tübingen	Nothing to declare
	Zih-Hua Fang	The German Center for Neurodegenerative Diseases	Nothing to declare
Ghana	Albert Akpalu	University of Ghana Medical School	Nothing to declare
	Vida Obese	Queen Square Institute of Neurology	Nothing to declare
Greece	Georgia Xiromerisiou	University of Thessaly	Nothing to declare
	Georgios Hadjigorgiou	University of Thessaly	Nothing to declare
	Ioannis Dagklis	Aristotle University of Thessaloniki	Nothing to declare

	Ioannis Tarnanas	Ionian University	Nothing to declare
	Leonidas Stefanis	Biomedical research Foundation of the Academy of Athens	PPMI2 (funded by MJFF), ALAMEDA (H2020 grant), funding by HFRI
	Maria Stamelou	Diagnostic and Therapeutic Centre HYGEIA Hospital	Nothing to declare
	Efthymios Dadiotis	University of Thessaly	Nothing to declare
Honduras	Alex Medina	Hospital San Felipe	Nothing to declare
Hong Kong	Germaine Hiu-Fai Chan	Queen Elizabeth Hospital	Nothing to declare
	Nancy Ip	Hong Kong University of Science and Technology	Nothing to declare
	Nelson Yuk-Fai Cheung	Queen Elizabeth Hospital	Nothing to declare
	Phillip Chan	Hong Kong University of Science and Technology	Nothing to declare
India	Asha Kishore	Aster Medcity	Michael J Fox Foundation
	Divya KP	Sree Chitra Tirunal Institute for Medical Sciences and Technology	Nothing to declare
	Pramod Pal	National Institute of Mental Health & Neurosciences	Nothing to declare
	Prashanth Lingappa Kukkle	Manipal Hospital	Nothing to declare
	Roopa Rajan	All India Institute of Medical Sciences	Nothing to declare
	Rupam Borgohain	Nizam's Institute Of Medical Sciences	Nothing to declare
Italy	Andrea Quattrone	University "Magna Graecia" of Catanzaro	Nothing to declare
	Enza Maria Valente	University of Pavia	Nothing to declare
	Lucilla Parnetti	University of Perugia	Nothing to declare
	Micol Avenali	University of Pavia	Nothing to declare
	Tommaso Schirinzi	University of Rome Tor Vergata	Nothing to declare
Japan	Manabu Funayama	Juntendo University	Nothing to declare
	Nobutaka Hattori	Juntendo University faculty of medicine	Nothing to declare

	Tomotaka Shiraishi	Jikei University School of Medicine	Nothing to declare
Kazakhstan	Altynay Karimova	Institute of Neurology and Neurorehabilitation	Nothing to declare
	Rauan Kaiyrzhanov	University College London	Nothing to declare
	Gulnaz Kaishibayeva	Institute of neurology and neurorehabilitation named after Smagul Kaishibayev	Nothing to declare
Luxembourg	Rejko Krüger	University of Luxembourg	Nothing to declare
Malaysia	Ai Huey Tan	University of Malaya	Nothing to declare
	Azlina Ahmad-Annuar	University of Malaya	Nothing to declare
	Mohamed Ibrahim Norlinah	Universiti Kebangsaan Malaysia	Nothing to declare
	Nor Azian Abdul Murad	UKM Medical Molecular Biology Institute (UMBI)	Nothing to declare
	Norlinah Mohamed Ibrahim	Universiti Kebangsaan Malaysia Medical Centre	Nothing to declare
	Shahrul Azmin	Universiti Kebangsaan Malaysia Medical Centre	Nothing to declare
	Shen-Yang Lim	University of Malaya	Nothing to declare
	Wael My Mohamed	International Islamic University	Nothing to declare
	Yi Wen	University of Malaya	Nothing to declare
Mexico	Daniel Martinez-Ramirez	Tecnologico de Monterrey	Nothing to declare
	Mayela Rodriguez-Violante	Instituto Nacional de Neurologia y Neurocirugia	Nothing to declare
	Paula Reyes-Pérez	Universidad Nacional Autónoma de México	Nothing to declare
Nepal	Rajeev Ojha	Tribhuvan University	Nothing to declare
New Zealand	Tim Anderson	University of Otago	Health Research Council of New Zealand; MBIE (NZ), Neurological Foundation of New Zealand; University of Otago

	Toni Pitcher	University of Otago	Funding - Health Research Council of New Zealand
Nigeria	Njideka Okubadejo	University of Lagos	Michael J Fox Foundation; Tertiary Education Trust Fund (TETFUND) National Research Fund
	Olaitan Okunoye	University College London	Nothing to declare
	Oluwadamilola Ojo	College of Medicine of the University of Lagos	Nothing to declare
Norway	Jan O. Aasly	Norwegian University of Science and Technology	Nothing to declare
	Lasse Pihlstrøm	Oslo University Hospital	Southeastern Regional Health Authority, Norway
	Manuela Tan	Oslo University Hospital	My postdoctoral position is funded by a grant to Dr Lasse Pihlstrøm from the Southern and Eastern Norway Regional Health Authority (Helse Sør-Ost). I have also received funding as a co-PI for a small grant from the Michael J Fox Foundation
Pakistan	Shoaib ur Rehman	University of science and Technology Bannu	Nothing to declare
Peru	Miguel Inca-Martinez	Cleveland Clinic	Nothing to declare
	Mario Cornejo-Olivas	Instituto Nacional de Ciencias Neurologicas	Nothing to declare
Puerto Rico	Angel Vinuela	University of Puerto Rico	Nothing to declare
Russia	Elena Iakovenko	Research Center of Neurology	Nothing to declare
Saudi Arabia	Bashayer Almubarak	King Faisal Specialist Hospital and Research Center	Nothing to declare
	Muhammad Umair	King Abdullah International Medical Research Center	Nothing to declare
Singapore	Jia Nee Foo	Nanyang Technological University	Singapore National Medical Research Council (MOH-000559)
South Africa	Jonathan Carr	University of Stellenbosch	Nothing to declare

	Soraya Bardien	Stellenbosch University	This work is based on the research supported in part by the South African Medical Research Council [Self-Initiated Research Grant]; the National Research Foundation of South Africa [Grant Number 129249]; and the South African Medical Research Council/Stellenbosch University Genomics of Brain Disorders Research Unit, Cape Town, South Africa
South Korea	Beomseok Jeon	Seoul National University Hospital	Nothing to declare
	Yun Joong Kim	Yongin Severance Hospital	Nothing to declare
Spain	Janet Hoenicka	Institut de Recerca Sant Joan de Deu	Fondo de Investigación Sanitaria, Instituto Salud Carlos III, Grant PI019/00126
	Maria Teresa Periñan	Instituto de Biomedicina de Sevilla	Nothing to declare
	Pau Pastor	University Hospital Germans Trias i Pujol	Nothing to declare
Sudan	Sarah El-Sadig	Faculty of medicine university of Khartoum	Nothing to declare
Taiwan	Chin-Hsien Lin	National Taiwan University Hospital	Nothing to declare
	Hsiu-Chuan Wu	Chang Gung Memorial Hospital	Nothing to declare
	Pin-Jui Kung	Genome and systems biology degree program	Nothing to declare
	Ruey-meei Wu	National Taiwan University Hospital	I have funding from 1. Minister of Science and Technology, Taiwan Government; 2. National Taiwan University, 3 Parkinson foundation, USA, 4. Michael J Fox Foundation.
	Serena Wu	Chang Gung University	Nothing to declare
	Yihru Wu	Chang Gung Memorial Hospital	Nothing to declare
Tunisia	Rim Amouri	National Institute Mongi Ben Hamida of Neurology	Nothing to declare

	Samia Ben Sassi	Mongi Ben Hmida National Institute of Neurology	Nothing to declare
Turkey	A. Nazlı Başak	Koç University	Kirac Foundation and Koc Univ.
	Gencer Genc	Şişli Etfal Training and Research Hospital	Nothing to declare
	Nazli Basak	Koc University	Nothing to declare
	Özgür Öztop Çakmak	Koç University	Nothing to declare
	Sibel Ertan	Koç University Medical School	Nothing to declare
United Kingdom	Alastair Noyce	Queen Mary University of London	Prof. Noyce reports grants from Parkinson's UK, Barts Charity, Cure Parkinson’s, NIHR, Innovate UK, Virginia Keiley benefaction, Alchemab, Aligning Science Across Parkinson’s and Michael J Fox Foundation. Consultancy and personal fees from Astra Zeneca, AbbVie, Profile, Roche, Biogen, UCB, Bial, Charco Neurotech, uMedeor and Britannia.
	Alejandro Martínez-Carrasco	University College London	GP2
	Camille Carroll	University of Plymouth	C Carroll receives salary from University of Plymouth, University Hospitals Plymouth NHS Trust and National Institute of Health Research; she has received advisory, consulting, and/or lecture fees from AbbVie, Bial, Lundbeck, Global Kinetics, Britannia and Medscape, and research funding from Parkinson's UK, Edmond J Safra Foundation, National Institute of Health Research and Cure Parkinson’s
	Claire Bale	Parkinson's UK	Nothing to declare
	Clodagh Towns	University College London	Nothing to declare
	Ellie Stafford	University College London	Nothing to declare
	Henry Houlden	University College London	Nothing to declare

	Huw R Morris	University College London	Dr Morris is employed by UCL. In the last 12 months he reports paid consultancy from Roche and Amylyx ; lecture fees/honoraria - BMJ, Kyowa Kirin, Movement Disorders Society. Research Grants from Parkinson's UK, Cure Parkinson's Trust, PSP Association, CBD Solutions, Drake Foundation, Medical Research Council, Michael J Fox Foundation. Dr Morris is a co-applicant on a patent application related to C9ORF72 - Method for diagnosing a neurodegenerative disease (PCT/GB2012/052140)
	John Hardy	University College London	Nothing to declare
	Joseph Callanan	Queen Mary University of London	Nothing to declare
	Kin Ying Mok	University College London	Nothing to declare
	Mie Rizig	University College London	Nothing to declare
	Nicholas Wood	University College London	ASAP- CRN
	Nigel Williams	Cardiff University	Parkinson's UK
	Patrick Alfryn Lewis	Royal Veterinary College University of London	MJFF, UKRI (BBSRC, EPSRC), Parkinson's UK, ASAP research network network
	Rimona Weil	University College London	Nothing to declare
	Simona Jasaitye	University College London	Nothing to declare
	Simon Stott	Cure Parkinson's	Employee of Cure Parkinson's
	Sumit Dey	Queen Mary University of London	Nothing to declare
USA	Alberto Espay	University of Cincinnati	Nothing to declare
	Alyssa O'Grady	The Michael J. Fox Foundation for Parkinson's Research	Nothing to declare
	Andrew B Singleton	National Institute on Aging	Michael J Fox Foundation for Parkinson's disease Research. Aligning Science Across Parkinson's Initiative

	Andrew K. Sobering	Augusta University / University of Georgia Medical Partnership	Nothing to declare
	Bernadette Siddiqi	The Michael J. Fox Foundation for Parkinson's Research	Nothing to declare
	Bradford Casey	The Michael J Fox Foundation for Parkinson's Research	Nothing to declare
	Brian Fiske	The Michael J. Fox Foundation for Parkinson's Research	Nothing to declare
	Cabell Jonas	Mid-Atlantic Permanente Medical Group / Kaiser Permanente Mid-Atlantic States	Nothing to declare
	Caroline B. Pantazis	National Institutes of Health	Nothing to declare
	Claire Wegel	Indiana University	Nothing to declare
	Cornelis Blauwendraat	National Institutes of Health	Nothing to declare
	Dan Vitale	National Institutes of Health	Nothing to declare
	Deborah Hall	Rush University	Nothing to declare
	Dena Hernandez	National Institutes of Health	Nothing to declare
	Ejaz Shiamim	Kaiser Permanente	Nothing to declare
	Ekemini Riley	Coalition for Aligning Science	Nothing to declare
	Faraz Faghri	National Institutes of Health	F.F.'s participation in this research was supported in part by the Intramural Research Program of the NIH, National Institute on Aging (NIA), National Institutes of Health, Department of Health and Human Services; project number ZO1 AG000535, as well as the National Institute of Neurological Disorders and Stroke. F.F.'s participation in this project was part of a competitive contract awarded to Data Tecnica International LLC by the National Institutes of Health to support open science research.

	Hampton Leonard	National Institute on Aging/National Institutes of Health	H.L.L is supported by a competitive contract awarded to Data Tecnica International LLC by the National Institutes of Health to support open science research
	Hirotaka Iwaki	Data Tecnica International	Nothing to declare
	Honglei Chen	Michigan State University	NIH/DoD/Parkinson Foundation/MSU Foundation/Gibby vs. Parky Foundation - No COI to disclose
	Ignacio F. Mata	Cleveland Clinic	Funding from MJFF and NIH
	Ignacio Juan Keller Sarmiento	Northwestern University	Nothing to declare
	Jared Williamson	Kaiser Permanente	Nothing to declare
	Jonggeol Jeff Kim	National Institutes of Health	Nothing to declare
	Joseph Jankovic	Baylor College of Medicine	Nothing to declare
	Joshua Shulman	Baylor College of Medicine / Texas Children's Hospital	Collection of samples and data at Baylor College of Medicine was supported by the Huffington Foundation.
	Justin C. Solle	The Michael J. Fox Foundation for Parkinson's Research	Nothing to declare
	Kaileigh Murphy	Operations and Compliance	Nothing to declare
	Karen Nuytemans	University of Miami Miller School of Medicine	This work has been supported by the American Parkinson Disease Association and the Margaret Q. Landenberger Research Foundation.
	Karl Kieburtz	Beth Israel Deaconess Medical Center	Nothing to declare
	Kenneth Marek	Institute for Neurodegenerative Disorders	Consultant for Michael J Fox Foundation, GE Healthcare, Roche, UCB, BIAL, Denali, Takeda, , Cerapsir, UCB, Biohaven, Neuron23, Aprinoia, Astellas, Calico, Genentech, Invicro
	Lana M. Chahine	University of Pittsburgh	Nothing to declare

	Laurel Screven	National Institute on Aging	Nothing to declare
	Lisa Shulman	University of Maryland	Nothing to declare
	Luca Marsili	University of Cincinnati	Nothing to declare
	Maggie Kuhl	The Michael J. Fox Foundation for Parkinson's Research	Nothing to declare
	Marissa Dean	University of Alabama at Birmingham	Dr. Dean is an investigator in studies funded by Abbvie, Inc., Hoffmann-La Roche, CHDI Foundation, Inc., Annexon, Inc., Retrophin, Inc, Neurocrine Biosciences, UniQure Biopharma B.V., Praxis Precision Medicines, Neuraly, Inc., Michael J. Fox Foundation for Parkinson's Research, and US Army Medical Research and Materiel Command (grant#W81XWH-18-1-0508). In addition, Dr. Dean receives support through the Huntington's Disease Society of American Centers of Excellence program.
	Mary B Makarious	National Institutes of Health	Nothing to declare
	Mike A. Nalls	National Institutes of Health	M.A.N.'s participation in this project was part of a competitive contract awarded to Data Tecnica International LLC by the National Institutes of Health to support open science research. M.A.N. also currently serves as an advisor for Clover Therapeutics and Neuron23 Inc.
	Niccolò Emanuele Mencacci	Northwestern University	Nothing to declare
	Roy Alcalay	Columbia University	Dr. Alcalay is funded by the Michael J. Fox Foundation and the Parkinson's Foundation. He received consultation fees from Avrobio, Caraway, GSK, Merck, Sanofi, Ono Therapeutics and Takeda
	Ruqaya Murtadha	National Institute on Aging	Nothing to declare

	Sara Bandres Ciga	National Institutes of Health	Nothing to declare
	Schuyler Fox	Michael J. Fox Foundation for Parkinson's Research	Nothing to declare
	Sohini Chowdhury	The Michael J. Fox Foundation for Parkinson's Research	Nothing to declare
	Steven Lubbe	Northwestern University	Nothing to declare
	Tao Xie	University of Chicago	Nothing to declare
	Tatiana Foroud	Indiana University School of Medicine	Michael J. Fox Foundation
	Thomas Beach	Sun Health Research Institution	Nothing to declare
	Todd Sherer	The Michael J Fox Foundation for Parkinson's Research	Nothing to declare
	Yared Z. Zewde	University of California San Francisco	Nothing to declare
	Yeajin Song	National Institutes of Health	Nothing to declare
Vietnam	Duan Nguyen	Hue University	Nothing to declare
	Toan Nguyen	Hue University	Nothing to declare
Zambia	Masharip Atadzhanov	University of Zambia	Nothing to declare
